# Epigenetics of discordant monozygotic twins: implications for disease

**DOI:** 10.1186/s13073-014-0060-z

**Published:** 2014-07-31

**Authors:** Juan E Castillo-Fernandez, Tim D Spector, Jordana T Bell

**Affiliations:** Department of Twin Research and Genetic Epidemiology, Kings College London, London, SE1 7EH UK

## Abstract

Monozygotic (MZ) twins share nearly all of their genetic variants and many similar environments before and after birth. However, they can also show phenotypic discordance for a wide range of traits. Differences at the epigenetic level may account for such discordances. It is well established that epigenetic states can contribute to phenotypic variation, including disease. Epigenetic states are dynamic and potentially reversible marks involved in gene regulation, which can be influenced by genetics, environment, and stochastic events. Here, we review advances in epigenetic studies of discordant MZ twins, focusing on disease. The study of epigenetics and disease using discordant MZ twins offers the opportunity to control for many potential confounders encountered in general population studies, such as differences in genetic background, early-life environmental exposure, age, gender, and cohort effects. Recently, analysis of disease-discordant MZ twins has been successfully used to study epigenetic mechanisms in aging, cancer, autoimmune disease, psychiatric, neurological, and multiple other traits. Epigenetic aberrations have been found in a range of phenotypes, and challenges have been identified, including sampling time, tissue specificity, validation, and replication. The results have relevance for personalized medicine approaches, including the identification of prognostic, diagnostic, and therapeutic targets. The findings also help to identify epigenetic markers of environmental risk and molecular mechanisms involved in disease and disease progression, which have implications both for understanding disease and for future medical research.

## Introduction

Epigenetics emerged during the first half of the 20th century as the study of biological mechanisms involved in embryonic development and cell differentiation [1]. More recently, it has been defined as the study of nuclear inheritance through cell division that is not based on differences in DNA sequence [2] and includes any mechanism that alters gene expression without altering DNA sequence. DNA methylation of cytosines at CpG dinucleotides was proposed as a mechanism of mammalian gene regulation in 1975 [3,4], and as it is the best studied epigenetic mechanism in human populations we will concentrate on it in this review. Typically, CpG methylation at the 5' regions of genes reduces gene expression. This downregulation is a result of either the inability of specific transcription factors to bind methylated CpGs or the recruitment of methyl-CpG-binding proteins (such as MeCP) with transcription repression activity [5-7]. Conversely, in gene body coding regions, patterns of high levels of methylation have been found in transcriptionally active genes [8]. Histone modifications, which are the next most studied epigenetic mark, are very diverse and may include acetylation, methylation, phosphorylation, ubiquitination, ADP-ribosylation, and others. It has been proposed that combinatorial modifications at selected residues trigger specific gene expression activity [9]. Less studied epigenetic regulators include histone variants [10,11], ATP-dependent chromatin remodeling complexes [12], and non-coding RNAs [13].

Apart from its key role in developmental biology, epigenetics has recently become relevant to epidemiology because it offers the promise of unraveling the biological mechanisms underlying disease and has potential as a biomarker of disease or of disease progression. In 1983, Feinberg and Vogelstein [14] reported epigenetic alterations of the human growth hormone and γ-globin genes in colon cancer patients. Since then, epigenetic alterations have been reported in many other types of cancer [15], autoimmune diseases [16], diabetes [17], Alzheimer's disease [18], Parkinson's disease [19], asthma [20], and multiple other human complex traits [21-23]. The majority of reported associations between epigenetic changes and phenotypic variation were observed in population samples of unrelated individuals. However, a number of studies have also explored epigenetic profiles in twins during normal development, aging, and in the context of disease, using disease-discordant MZ twins.

**Figure 1:**
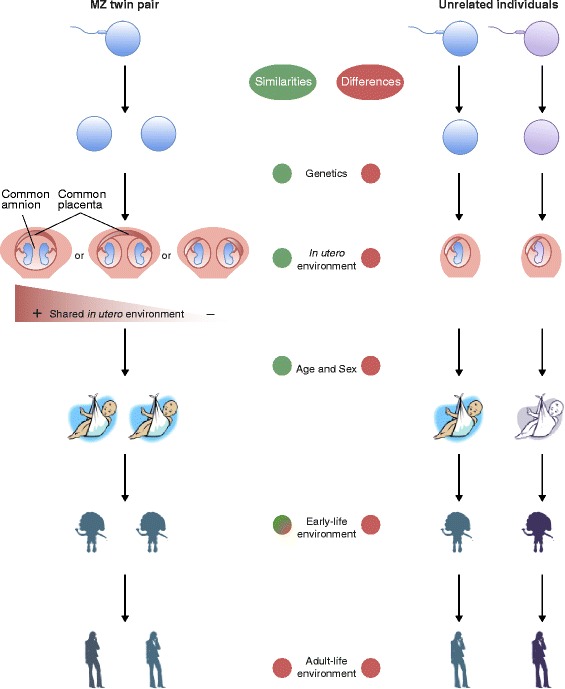
**Shared and non-shared potential epigenetic confounding factors throughout the lifetime of MZ twins and unrelated individuals.** MZ twins, in contrast with unrelated individuals, share most genetic variants, as well as similar prenatal and early-life environments. The *in utero* environment is also shared by MZ twins, although to different degrees: they can have a shared placenta and amnion (monochorionic monoamniotic, left picture), a shared placenta but different amnion (monochorionic diamniotic, middle), or a different placenta and amnion (dichorionic diamniotic, right). Epigenetic studies using MZ twins allow the control of most genetic, maternal, environmental, and cohort effects.

Epigenetic disease studies can particularly benefit from the unique study design of disease-discordant MZ twins. The use of MZ twins allows us to study the role of epigenetics in disease by controlling for many potential confounders, such as genetic factors, age, gender, maternal effects, cohort effects, and most *in utero* and environmental influences (Figure 1). Both population-based and twin-based epigenetic studies are susceptible to bias from potential unobserved confounders, and require replication to minimize false positive findings. Here, we discuss the benefits, challenges (Box 1) and limitations (Box 2) of epigenetic studies using disease-discordant monozygotic (MZ) twins (also called identical twins), and we review recent findings and their implications for medical research.

## Monozygotic twins and epigenetics

MZ twins arise from the same single cell and therefore share almost all of their genetic variants (Figure 1). Nevertheless, these isogenic individuals are not completely identical, but show phenotypic discordance for many traits from birth weight to a range of complex diseases [24]. Proband-wise concordance rate estimates show that MZ twins are relatively often discordant for most common complex diseases, such as type 1 diabetes (T1D; 61%) [25], type 2 diabetes (41%) [26], autism (58 to 60%) [27], schizophrenia (58%) [28], and different types of cancer (0 to 16%) [29]. This observation supports the finding that for many complex traits, genotype alone may not fully determine phenotypic variation, and the interplay between genes and environment needs to be considered. Epigenetics has been proposed to be one of the main mediators of this interaction [30].

Fraga *et al*. [31] first demonstrated that MZ twin pairs exhibit epigenetic differences by comparing total content of methylcytosine and histone acetylation levels of peripheral lymphocytes. Twin pairs who were older, spent less time together, or had more dissimilar health or medical histories showed greater differences in both types of epigenetic marks. In 2009, Kaminsky *et al*. [32] confirmed the presence of DNA methylation differences between co-twins across three different tissues (white blood cells (WBCs), buccal epithelial cells, and gut biopsies) in an age- and sex-matched twin sample. They also observed greater similarity between DNA methylation profiles within MZ twins than within dizygotic (DZ) twins. Furthermore, they proposed a functional stratification of the epigenome based on the finding of lower variability between co-twins in CpG islands and promoters than in all non-CpG island loci. Similar region-specific variation in DNA methylation was found from profiling of the major histocompatibility complex (MHC) in CD4^+^ lymphocytes of 49 Norwegian MZ twins [33]. CpG islands, 5' regions, and conserved non-coding regions were less variable than CpG-poor regions within twins. The reported epigenetic differences between MZ co-twins appear to be established early on in life, as they were also observed in the neonatal epigenome in a longitudinal genome-wide study of DNA methylation that profiled 10 MZ and 5 DZ twin pairs at birth and at 18 months [34]. Interestingly, in the early years epigenetic profiles tended to diverge, converge, or remain discordant in a twin-pair-specific manner, showing high variability compared with adults.

The process of establishment and maintenance of epigenetic marks may be one explanation for the epigenetic discordance between MZ co-twins. DNA methylation is laid down by DNA methyltransferases and inherited through cell division, and epimutations (differences in epigenetic marks) can arise during this process. The DNA methyltransferases (DNMTs) Dnmt3a and Dnmt3b are involved in *de novo* methylation, whereas Dnmt1 maintains the marks across cell division. Methylcytosines are not added during DNA replication; as a consequence, the newly synthesized strands will lack methylation marks and the DNA molecules would be methylated only on one strand. Dnmt1 uses this hemi-methylated molecule as a substrate and methylates the corresponding nucleotides on the complementary strand. Epimutations, which can be inherited through cell division, may occur as probabilistic errors in the correct function of these enzymes. For example, Dnmt1 skips 4 to 5% of methylation sites and shows *de novo* methylation activity near densely methylated regions [35].

Failure in the correct establishment of DNA methylation patterns has a key role in human genetic diseases known as imprinting disorders. Parent-of-origin-specific gene expression, known as genomic imprinting, is an epigenetic mechanism in which the imprinted gene is differentially methylated at the paternal and maternal alleles [36]. The region that controls the expression of an imprinted gene is known as an imprinting center. For example, methylation of the Prader-Willi imprinting center in chromosome 15q on the paternal allele causes Prader-Willi syndrome [37], while failure to methylate the same locus on the maternal allele causes Angelman syndrome [37], and loss of DNA methylation at the *KCNQ1OT1* gene on the maternal allele causes Beckwith-Wiedemann syndrome [38]. In two separate studies using twins discordant for Beckwith-Wiedemann syndrome, Weksberg *et al*. [39] and Bliek *et al*. [40] found that the affected twins (5 of 5 MZ twin pairs and 10 of 10 MZ twin pairs, respectively) had the epimutation. Both studies also emphasize the fact that there is an excess of discordant female MZ twins in Beckwith-Wiedemann syndrome patients (8% [39] and 2.5% [40]) compared with the general population (0.3 to 0.4%). This observation has suggested a link between epigenetic maintenance failures and the MZ twinning event, which has also previously been proposed by Bestor [41]. MZ twin discordance for other imprinting disorders has also been described, for example, in Silver-Russell syndrome [42,43], but in relatively small numbers.

Epigenetic differences in MZ twins may also arise as a result of environmental differences. MZ twins are exposed to different nutritional conditions related to the formation and vascularization of the placenta [44]. Because the intrauterine environment of MZ twins can differ, it is possible that intrauterine differences contribute to differences in epigenetic states (Box 2). DNA methylation profiles are less similar within pairs of monochorionic (shared placenta) MZ twins than within pairs of dichorionic (non-shared placenta) MZ twins [32,45]. This observation suggests that sharing a placenta may cause imbalanced *in utero* conditions and, consequently, more discordant epigenetic profiles. Another factor that may result in early-life epigenetic differences in twins is an unequal allocation of cells during the twinning event; however, not much detail is known about the precise mechanism of twinning. Environmental factors may also contribute to epigenetic differences in adult twins over time. Cigarette smoking is a known environmental modifier of DNA methylation. Smoking was first associated with differential DNA methylation in an adult population (50 to 60 years of age) at the *F2RL3* gene, which encodes coagulation factor II receptor-like 3 [46]. Since then, multiple studies have identified and replicated smoking-associated changes in DNA methylation at several genes, including the well replicated aryl hydrocarbon receptor repressor (*AHRR*) gene [47-49] and several other regions. Interestingly, at multiple smoking-associated sites DNA methylation levels of former smokers are generally similar to levels observed in non-smokers, which suggests that smoking cessation may allow methylation levels to revert back. In addition to smoking, many other environmental factors have been linked to changes in DNA methylation, although not in humans, including diet, exposure to environmental toxins, temperature changes, and others [50]. As adult lifestyles of twins diverge, it is likely that their epigenomes may too. Traditionally, MZ twin discordance has been attributed only to environmental differences, but stochastic events, which are difficult to measure, may also play a role.

Lastly, although MZ twins are often referred to as being genetically identical, post-zygotic mutation events can occur (Box 2). Somatic point mutations during early development occur with a frequency of 1.2 × 10^−7^ per base pair per twin pair [51]. Similarly, somatic copy number variations have been found in normal concordant and in disease-discordant MZ twin pairs [52]. The influence of genetic variation on epigenetic marks has been previously demonstrated by the association of specific genetic variants at single nucleotide polymorphisms with specific DNA methylation sites [53]; it is therefore possible that somatic mutations or copy number variations in MZ twins may lead to epimutations in a cell- and tissue-specific manner.

## Twin discordance for disease and environmental factors

Most complex phenotypes arise as a result of the interplay between genetics and environment. In epidemiology, it is of interest to determine what proportion of the phenotypic variance each of these factors can explain. Classical twin studies make use of MZ and DZ twins to decipher these influences. Because MZ twins are assumed to share almost 100% of their genetic variants, and DZ twins share, on average, only 50% of their variants, the difference in phenotype concordance levels between these two groups can indicate a genetic influence on the phenotype. Greater phenotype concordance in MZ twins would point to a higher contribution of genetics to the disease.

**Figure 2:**
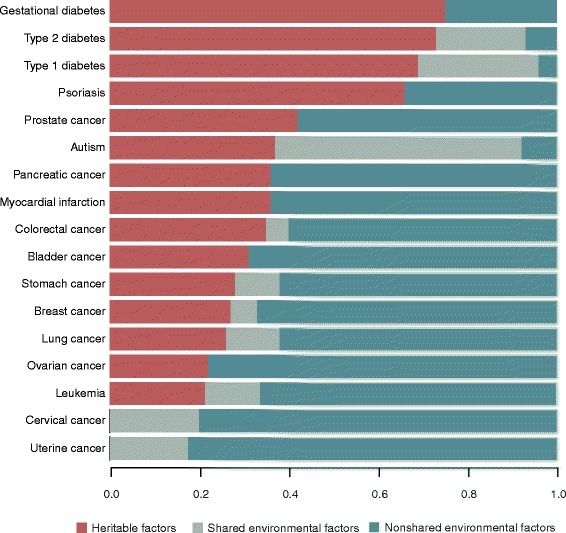
**Heritable and environmental factors contributing to disease.** For each complex disease we show the fraction of the phenotypic variance explained by heritable factors, shared environmental factors, and non-shared environmental factors. The estimates were obtained from published studies of stomach, colorectum, pancreas, lung, breast, cervical, uterine, ovary, prostate, bladder and leukemia cancers [29]; strict autism [27]; psoriasis [56]; myocardial infarction [57]; gestational diabetes, type 1 diabetes, and type 2 diabetes [25].

Heritability in the broad sense (*H*^2^) has been used to define the fraction of the total phenotypic variance (${\sigma}_P^2$) in a population that can be attributed to genetic variance (${\sigma}_G^2$) $\left[{H}^2=\frac{\sigma_G^2}{\sigma_P^2}\right]$. Genetic variance can further be partitioned into the variance attributed to additive (${\sigma}_A^2$), dominant (${\sigma}_D^2$), and epistatic (${\sigma}_I^2$) effects. This partition gives origin to the definition of heritability (*h*^2^) in the narrow sense, which only considers the fraction of the total phenotypic variance attributed to additive genetic effects $\left[{h}^2=\frac{\sigma_A^2}{\sigma_P^2}\right]$ [54]. The rest of the variation that is not attributed to genetic effects is attributed to environmental influences, which can be divided into shared and unique environmental influences and random error. The classical twin estimate of heritability, known as *h*^2^, is defined as twice the difference between MZ and DZ intra-pair correlation coefficients (ICCs) of the trait of interest [*h*^2^ = 2(*ICC*_*MZ*_ − *ICC*_*DZ*_)] [55]. The twin model also assumes that MZ and DZ twins equally share the environment and that a fraction of the correlation is due to the shared environmental influences. The fraction corresponding to shared environmental effects is estimated as the difference between the total correlation and the heritability estimate. Finally, the missing variation is attributed to unique environmental effects plus error. Figure 2 shows these proportions estimated by different studies for several diseases, including diabetes, psoriasis, autism, myocardial infarction, and different types of cancer.

Heritabilities can be calculated for particular epigenetic variants at specific epigenetic loci by treating them like phenotypes. Kaminsky *et al*. [32] estimated the locus-specific DNA methylation ICC differences (*ICC*_*MZ*_ − *ICC*_*DZ*_) of 40 age- and sex-matched MZ and DZ twin pairs in buccal epithelial cells and WBCs at about 6,000 loci. The differences were significant in both tissues, but greater in buccal epithelial cells (mean *ICC*_*MZ*_ − *ICC*_*DZ*_ = 0.15 ± 0.0039, *P* = 1.2 × 10^− 294^) than in WBCs (mean *ICC*_*MZ*_ − *ICC*_*DZ*_ = 0.0073 ± 0.0034, *P* = 0.044), showing that at a proportion of sites in the genome DNA methylation levels show evidence for heritability. Gervin *et al*. [33] estimated heritabilities of DNA methylation across CpG sites in the human MHC in CD4^+^ lymphocytes using bisulfite sequencing. Their relatively low heritability estimates (2 to 16%) suggested that DNA methylation variation in the MHC is mostly due to environmental factors. A subsequent larger study using whole blood samples from 172 female twins examined 26,690 CpG sites on the Illumina Infinium HumanMethylation27 BeadChip (Illumina 27 K) that includes CpG sites only at promoter regions, and revealed slightly higher DNA methylation heritability per CpG site, with an average of 18.2% across the genome [58]. DNA methylation heritability was supported by the significant association of genetic variants with 6.3% of the probes. More recently, Gordon *et al*. [45], using the Illumina 27 K array, estimated heritabilities on three different tissues at birth: human umbilical vascular endothelial cells (HUVECs), cord blood mononuclear cells, and placenta. The results suggested a tissue-specific effect on heritability, in which only three of the top 5% most heritable sites were shared across all three tissues. However, given the low coverage of the Illumina 27 K array, it may be difficult to generalize these results. Decomposition of the epigenetic variance into genetic, shared environmental, and non-shared environmental effects in different twin studies so far show that, overall, non-shared environmental and stochastic factors may have a greater effect on methylation sites than genetic and shared environmental effects [34,45]. However, many of these studies rely on array technologies that may be subject to technical noise that may affect the accurate measurement of DNA methylation, particularly subtle changes [59]. Heritability estimates may also be confounded by the presence of cellular heterogeneity in the sample, which may introduce DNA methylation differences, for example, as shown in saliva samples [60], and to a lesser extent in buccal cells [34].

A different approach to study genetic effects on epigenetics focused on chromatin states, which are key to epigenetic mechanisms [61]. The authors explored discordant chromatin accessibility states associated with genetic variants in lymphoblastoid cells of 36 MZ twin pairs. Within-pair differences in chromatin accessibility were increased in the presence of somatic mutations and had a significant effect on gene expression discordance. Furthermore, quantitative trait locus mapping revealed chromatin accessibility differences at 1,325 loci associated with genotypic variants at a false discovery rate of 0.01. This integrative analysis using MZ twins shows how genetic factors affect chromatin states, potentially through epigenetic mechanisms, and ultimately gene expression.

Discordant lifestyles are obvious potential triggers for disease discordance. Epidemiological studies using disease-discordant MZ twins have identified environmental or lifestyle factors that increase or decrease the risk of developing a disease. Increased risk for basal cell carcinoma of the skin was associated with smoking status in females and decreased risk with outdoor work [62], and because smoking is strongly linked to DNA methylation changes at multiple genes [49], DNA methylation is a potential mediator of the molecular impacts of smoking on the disease in this case. Additional findings include moderate alcohol consumption, which associates positively with bone mineral density [63]. Furthermore, a study of MZ twins from the Older Australian Twins Study revealed that cognitive and social activity was associated with performance on some cognitive tasks [64]. Finally, habitual physical activity during adulthood enlarges the distal aorta and iliac and femoral artery diameters according to a study that included six middle-aged MZ twins with 32 years of discordance for physical activity [65]. Although the link between most lifestyle changes and specific epigenetic marks that may lead to disease is still unknown, MZ twins provide a good system to identify environmentally associated DNA methylation modifications and study their role in disease.

Low birth weight is an environmentally associated phenotype that has become of interest in epigenetics and disease because epidemiological studies have associated it with increased rates of coronary heart disease, stroke, hypertension, and type 2 diabetes [66]. Epigenetic studies have taken advantage of the birth weight differences that occur between MZ twin siblings, due to the competition for resources during their intrauterine development, to study methylation differences associated with birth weight. Gordon *et al.* [45] identified differential methylation of a gene with vascular function, *APOLD1*, as being associated with birth weight after adjustment for multiple testing using umbilical vascular endothelial cells of 14 MZ twin pairs. A later study was conducted by Souren *et al.* [60] using DNA extracted from saliva of 17 monochorionic MZ twin pairs with relative birth weight differences greater than 20%. At nominal significance and with absolute methylation differences greater than 0.05, 45 CpG sites had an uncorrected *P*-value <0.01 and absolute methylation differences greater than 0.05, but validation of those loci by bisulfite sequencing showed that the methylation differences may be due to technical variation.

## Epigenetic studies of twins and disease

Multiple studies have explored the epigenetic changes in twins discordant for a range of diseases (Table 1). Here, we focus predominantly on recent genome-wide efforts across four major types of disease.

**Table 1:** Epigenetic studies using discordant MZ twins*

**Phenotype**	**Assay**	**Tissue (cell heterogeneity correction)** ^**†**^	**Major findings**	**Identification cohort**	**Validation/ replication**	**References** ^**‡**^
Adolescent depression	Illumina 450 K	Buccal cells (no correction)	Two differentially methylated positions reproducible in brain	18 discordant pairs of MZ twins	Validation: bisulfite pyrosequencing	[67]
Alzheimer’s disease	Immunohistochemistry for 5-methylcytosine	Temporal neocortex (no correction)	Significantly reduced levels of DNA methylation	1 discordant MZ twin pair		[68]
Autism spectrum disorders	Custom array^**§**^	Lymphoblastoid cells (no correction)	73 differentially methylated CpG islands	3 pairs of male discordant MZ twin pairs	Validation: bisulfite sequencing and methylation-specific PCR	[69]
Autism spectrum disorders	Illumina 27 K	Whole blood (no correction)	Differentially methylated regions in genes already mentioned in the literature	34 discordant and 5 concordant MZ twin pairs for ASD or a related trait	Validation: bisulfite pyrosequencing	[70]
Bipolar disorder	Illumina 27 K	Whole blood (no correction)	Hypomethylation in the upstream region of *GPR24*	11 discordant MZ twin pairs		[71]
Bipolar disorder	MS-RDA	Lymphoblastoid cells (no correction)	Hypomethylation of *PPIEL*	1 discordant MZ twin pair, 23 unrelated cases, and 18 unrelated controls	Validation: bisulfite pyrosequencing	[72]
Birth weight	Illumina 450 K	Saliva (correction for buccal epithelium and leukocyte subtypes)	45 differentially methylated CpG sites	17 discordant monochorionic MZ female twin pairs	Validation: bisulfite sequencing	[60]
Breast cancer	Illumina 450 K	Whole blood (no correction)	*DOK7* as a candidate for blood-based cancer diagnosis	15 discordant MZ twin pairs	Validation: bisulfite pyrosequencing of 21 discordant MZ twin pairs	[73]
Caudal duplication anomaly	Targeted bisulfite sequencing	PBMCs and buccal cells (no correction)	Hypermethylation of the *AXIN1* promoter in PBMCs, but not in buccal epithelia	1 discordant MZ twin pair		[74]
Childhood leukemia and secondary thyroid carcinoma	Targeted bisulfite pyrosequencing	Primary skin fibroblasts (no correction)	Increased methylation of *BRCA1*	1 discordant MZ twin pair		[75]
Congenital renal agenesis	RRBS	Whole blood (no correction)	514 DMRs in 25 genes, including 6 related to organ development	1 discordant MZ twin pair		[76]
Major depressive disorder	MeDIP-seq	Whole blood (correction for lymphocytes, monocytes, neutrophils, and eosinophils)	Hypermethylation of a region within the *ZBTBT20* gene	50 discordant MZ twin pairs	Replication: MeDIP-seq in 356 unrelated case–control samples	[77]
Major psychosis	Illumina 27 K	Whole blood (no correction)	Hypomethylation in the promoter region of *ST6GALNAC1*	22 discordant MZ twin pairs	Replication: EpiTYPER in 45 post-mortem brain samples	[71]
Multiple sclerosis	RRBS	CD4^+^ lymphocytes (no correction)	No reproducible differences were detected	3 discordant MZ twin pairs		[78]
Pain sensitivity	MeDIP-seq	Whole blood (correction for lymphocytes, monocytes, neutrophils, and eosinophils)	Significant association signal in the promoter of the gene *TRPA1*	25 discordant MZ twin pairs	Replication: MeDIP-seq in 50 unrelated samples. Validation: bisulfite pyrosequencing and Illumina 450 k	[79]
Psoriasis	Illumina 27 K	CD4^+^ and CD8^+^ cells (no correction)	Correlation between DNA methylation differences and gene expression differences of genes involved in the immune response only in CD4^+^	CD4^+^: 17 discordant MZ twins pair; CD8^+^: 13 discordant MZ twins pairs		[80]
Schizophrenia	Illumina 27 K	Whole blood (no correction)	Hypermethylation in the upstream region of *PUS3*	11 discordant MZ twin pairs		[71]
Schizophrenia	Targeted bisulfite sequencing	Lymphocytes (no correction)	Patterns of methylation in the 5'-regulatory region of *DRD2* gene are closer between affected individuals	1 discordant and 1 concordant MZ twin pair		[81]
Systemic lupus erythematosus	Illumina GoldenGate Methylation Cancer Panel I	White blood cells (no correction)	49 differentially methylated genes potentially relevant in autoimmune inflammatory diseases processes	5 discordant MZ twin pairs	Validation: bisulfite sequencing	[82]
Type 1 diabetes	Illumina 27 K	CD14^+^ monocytes (no correction)	132 differentially methylated CpG sites	15 discordant MZ pairs	Validation: bisulfite pyrosequencing. Replication: Illumina 27 K of 4 twin pairs	[17]
Type 1 diabetes	Illumina 27 K	Immortalized B cell lines (no correction)	88 differentially methylated CpG sites	3 discordant and 6 concordant MZ twin pairs	Validation: bisulfite sequencing	[83]
Ulcerative colitis	Illumina 27 K and MeDIP-chip	Intestinal biopsies (no correction)	61 disease-associated genes	10 discordant MZ pairs	Validation: bisulfite pyrosequencing	[84]

*Abbreviations: ASD, autism spectrum disorders; DMR, differentially methylated region; MeDIP-chip, methylated DNA immunoprecipitation and array hybridization (Nimblegen custom 385 k Tiling Arrays in [84]); MS-RDA, methylation-sensitive representational difference analysis; MZ, monozygotic; PBMC, peripheral blood mononuclear cell; RRBS, reduced representation bisulfite sequencing.

^†^Tissue or cell sample used at discovery stage; cell heterogeneity correction approach is given in parentheses if relevant.

^‡^Effect sizes are described in the text.

^§^8.1 K CpG island microarrays (UHN Microarray Centre).

### Cancer

Many epigenetic aberrations have been identified in cancer. Two recent studies of cancer-discordant MZ twins [73,75] have provided interesting findings. High-resolution profiling of DNA methylation in whole blood samples from breast-cancer-discordant MZ twins led to the identification of *DOK7* hypermethylation as a blood-based epigenetic biomarker that can be traced years before tumor diagnosis [73]. *DOK7* encodes a docking protein that acts as a substrate and activator of receptor tyrosine kinase. Heyn *et al*. [73] used the Infinium HumanMethylation450 BeadChip (Illumina 450 K array) to profile 15 MZ twin pairs at about 485,000 CpG sites genome-wide, focusing on genes, including promoters and gene bodies, as well as intergenic regions. The authors identified 403 positions that were differentially methylated in breast cancer in whole blood from 15 MZ twin pairs discordant for the disease. Among these positions they found a hypermethylated site located in an alternative promoter of the *DOK7* gene with a methylation difference of only 2%. Further analysis revealed that the methylation signal spreads upstream of the promoter. Interestingly, the methylation of *DOK7* was also found in samples taken, on average, 4.7 years before tumor diagnosis, implying that the epigenetic change occurs early in the development of the disease. Although the consequences of an altered *DOK7* methylation status are unknown, these results indicate a promising role for *DOK7* as a blood-based biomarker and serve as a model for other larger studies. In addition to this finding, the authors [73] identified a breast cancer epigenetic signature that comprises 46 of the differentially methylated positions that can cluster samples according to the presence of cancer. Further study of the associated genes could give valuable clues to the pathogenesis of the disease.

Galetzka *et al*. [75] studied an interesting case that suggests that epigenetic mosaicism is the source of variation between one pair of MZ twins discordant for childhood leukemia and secondary thyroid carcinoma. Using bisulfite pyrosequencing of target genes known to be associated with cancer (*ATM*, *BRCA1*, *BRCA2*, *MLH1*, *RAD51C*, and *TP53*), they found differential methylation of *BRCA1* in skin fibroblasts and saliva. The difference in methylation levels in fibroblasts was 9% and in saliva was 7%. The methylation patterns of individual DNA molecules revealed epigenetic mosaicism in the affected twin, with 13% of her alleles exhibiting the epimutation. Although sequence variants have been linked to aberrant methylation patterns, no sequence alteration was found in the 5' regulatory region of the gene. Protein expression analysis demonstrated lower levels of BRCA1 in the affected twin, consistent with the idea that hypermethylation of tumor suppressor gene promoters is associated with gene silencing. Although the possibility of late somatic events as a consequence of treatment cannot be ruled out, the epigenetic mosaicism may have resulted from an epimutation during early embryonic stages that affected a subpopulation of cells. This study [75] highlights the importance of early embryonic stages during the development of some epigenetic marks and not just adult-life environmental factors.

MZ twins are also useful for identifying genetic factors linked to epigenetic mechanisms that may lead to cancer. The study of a pair of MZ twins discordant for *MLL* gene-rearranged leukemia helped to identify mutations in a histone H3 lysine36 methyltransferase, SETD2, that causes changes in the epigenetic state of the cell [85]. A different approach used two pairs of MZ twins with acute lymphoblastic leukemia to study the timing of genomic lesions that may lead to this disease [86]. Thus, the interplay between genetics and epigenetics can also be studied using MZ twins.

### Autoimmune disorders

One of the first genome-wide DNA methylation studies of autoimmune disease was performed by Javierre *et al*. [82], who explored three autoimmune diseases with overlapping clinical signs and symptoms: systemic lupus erythematosus (SLE), rheumatoid arthritis, and dermatomyositis. DNA methylation of WBCs was assayed with a platform designed for cancer analysis (GoldenGate Methylation Cancer Panel I) that included 1,505 CpG sites from 807 genes. The cohort included five discordant MZ twin pairs for each disease and 30 unrelated normal controls. The authors [82] found no differences in DNA methylation of the five MZ twin pairs for rheumatoid arthritis and dermatomyositis, but they found a set of 49 genes differentially methylated in SLE discordant twins with absolute mean methylation differences >0.1. Ontology analysis revealed that the differentially methylated regions (DMRs) were linked to genes enriched in functional processes related to autoimmune inflammatory diseases. Successful confirmation of the methylation differences at eight genes was carried out by pyrosequencing of additional related samples.

A more extensive recent epigenetic study of T1D used the Illumina 27 K array for genome-wide DNA methylation analysis of a specific subset of immune cells, monocytes, from 15 MZ twins discordant for T1D. The results revealed the presence of T1D-specific methylation variable positions in the T1D-affected co-twins, with effect sizes of small magnitude, from 0.13% to 6.6% [17]. The T1D-specific methylation variable positions were replicated in a set of four independent MZ pairs discordant for the disease. Furthermore, using samples from seven singletons before and after diagnosis, they demonstrated that methylation differences at these sites precede clinical diagnosis. DNA methylation changes associated with T1D were also observed by Stefan *et al.* [83] using the same array and immortalized B cell lines from only three T1D-discordant and six T1D-concordant MZ twin pairs. The authors identified 88 differentially methylated sites present in all three discordant pairs and, interestingly, six of the identified genes are known to be associated with the disease from genome-wide association studies. These findings in part overlap with the results of Rakyan *et al.* [17]: both reported differentially methylated positions in the MHC region. Replication of these findings is pending, but may contribute to understanding the role of epigenetic mechanisms in the etiology of T1D.

Using an integrative approach, Gervin *et al*. [80] combined genome-wide DNA methylation and gene expression profiles to study psoriasis in CD4^+^ cells from 17 discordant MZ twins and CD8^+^ cells from 13 discordant MZ twins pairs. The DNA methylation profiles were once again obtained using the Illumina 27 K array, and gene expression was explored using the Illumina HT-12 array. The independent methylation and expression analyses did not detect any significant difference in DNA methylation or expression, but the combined analysis revealed correlation between the co-twin DNA methylation difference and the log fold expression ratio of genes involved with immune response in CD4^+^ cells. These findings highlight important pathways for the study of the disease, but require further study with more extensive DNA methylation coverage, as well as replication in a larger cohort. Another integrative approach included the analysis of differentially methylated CpG sites characterized with the Illumina 27 K array, DMRs obtained by methylated DNA immunoprecipitation and array hybridization (MeDIP-chip), and differential gene expression interrogated with Affymetrix UG 133 Plus 2.0 arrays to study ulcerative colitis with intestinal biopsies of 10 discordant MZ twin pairs [84]. In total, 61 genes with differential expression and at least one differentially methylated position or region were defined as disease-associated. These integrative approaches allow the association of methylation with gene expression, but cannot determine causality. Different approaches are needed to establish whether DNA methylation is controlling gene expression.

### Psychiatric disorders

One early study of epigenetics in schizophrenia used lymphocytes of one discordant and one concordant pair of MZ twins to characterize the 5’ regulatory region of the dopamine D2 receptor gene (*DRD2*) through bisulfite sequencing [81]. The results showed that the epigenetic profile of the affected twin was more similar to the profile of the affected concordant twins than to the profile of their unaffected co-twin. The finding was of relevance for studying epigenetics and schizophrenia, but involved only a single gene.

Recent genome-wide epigenetic studies have identified interesting findings in major psychosis and depression. Dempster *et al*. [71] studied epigenetic changes in schizophrenia and bipolar disorder (BD) independently and as a major psychosis group. DNA methylation was assayed in blood samples from 22 MZ twin pairs discordant for schizophrenia or BD using the Illumina 27 K array. The strongest associated DMR in the schizophrenia group was found upstream of the tRNA pseudouridine synthase 3 gene *(PUS3*), in the BD group was upstream of the melanin-concentrating hormone receptor 1 gene (*GPR24*), and in the major psychosis group it was found in the promoter region of *ST6GALNAC1. ST6GALNAC1* encodes a protein involved in glycosylation and cell-cell interactions, and the identified epigenetic change overlapped a previously reported rare genomic duplication observed in schizophrenia. The reported effect size at this epigenetic variant was relatively modest: differences in methylation between affected and unaffected twins were, on average, less than 10%. Some of the top-ranked differentially methylated sites in the two groups were shared, but disorder-specific alterations were also observed with DNA methylation changes in opposite directions. This study also supported the idea of epimutations across tissues with the observation of hypomethylation of the *ST6GALNAC1* DMR in 4 out of 30 post-mortem brain tissue samples of affected individuals.

A different approach to study BD used methylation-sensitive representational difference analysis to characterize DNA methylation in lymphoblastoid cell lines of only one pair of discordant MZ twins [72]. One of the isolated differentially methylated fragments corresponded to the regulatory region of the pseudogene *PPIEL*. Its function is unknown, but a strong inverse correlation between the expression and DNA methylation levels of *PPIEL* was found.

Depression is another psychiatric disorder with interesting epigenetic findings. Using methylated DNA immunoprecipitation (MeDIP) with sequencing, Davies *et al*. [77] identified hypermethylation of a region within the *ZBTBT20* gene associated with major depressive disorder (MDD). *ZBTB20* has an important role in the developing hippocampus, a region previously implicated in the development of MDD. The discovery cohort included 50 MDD-discordant MZ twin pairs and the finding was replicated in a cohort of 356 unrelated case–control individuals. Although a previous study using the 450 K array was unable to observe significant DNA methylation differences using 12 MDD-discordant MZ twin pairs [87], differences in sample size and much greater genome coverage of the MeDIP-seq method in comparison with the 450 K array (which only covers 8.9% of dynamic CpGs [88]) may account for the differences. However, both studies reported a greater genome-wide methylation variance in the MDD-affected twins than in the unaffected siblings. This study is among the largest epigenetic twin studies published so far (50 MZ discordant twin pairs), with an independent replication sample, but lacks validation of the findings using an alternative DNA methylation detection technique.

Adolescent depression was also studied in 18 pairs of MZ twins with discordant scores on self-rated depression [67]. Buccal cells were characterized in this study because of their low heterogeneity compared with blood, and the DNA methylation assay was performed with the Illumina 450 K array. Two of the top 10 differentially methylated sites with methylation differences less than 10% were also found in post-mortem brain samples of patients with MDD. The methylation of these two sites shared across tissues has a potential use for development as biomarkers.

### Neurological diseases and pain

One of the earliest epigenetic studies using disease-discordant MZ twins explored caudal duplication anomaly [74], a condition characterized by duplication of organs in the caudal region. The authors used a candidate gene approach to examine DNA methylation at the promoter region of a gene implicated in these anomalies, *AXIN1.* Hypermethylation of the region was detected in the affected twin in peripheral blood mononuclear cells (PBMCs), but not in buccal cells. Because blood is mesodermal tissue whereas buccal cells are ectodermal, this result suggests that the epimutation occurred after the differentiation of the three germ layers and highlights the tissue-specific role of epigenetic marks.

The epigenetics of Alzheimer’s disease has also been studied, though with a limited sample size. An immunochemistry assay was performed on the temporal neocortex of a pair of discordant MZ twins [68]. With this technique it was only possible to establish that the affected twin possessed reduced levels of DNA methylation in temporal neocortex, but not in other parts of the brain. This study highlights the importance of tissue selection in epigenetic studies and the difficulty of finding markers shared across tissues.

Subsequent genome-wide efforts included a small epigenetic twin study of multiple sclerosis, which is a demyelinating disease that causes neurodegeneration. Baranzini *et al*. [78] compared the genome-wide methylation of three MZ twin pairs discordant for multiple sclerosis using reduced representation bisulfite sequencing. CpG methylation differences were few, and no differential methylation was common in at least two pairs. This was the first attempt to study this disease in discordant MZ twins at this level of resolution, but the results were based on only three twin pairs. A larger sample size is needed to have reasonable statistical power to detect epigenetic changes. Furthermore, methylation was interrogated by a sequencing technology that only captures 11.5% of the dynamic CpG sites [88].

Autism is a neurodevelopmental disorder with considerable twin discordance. One early approach with only three pairs of discordant MZ twins allowed the identification of 73 differentially methylated CpG islands in lymphoblastoid cell lines [69]. Methylation was interrogated with the 8.1 K CpG island microarray (UHN Microarray Center). The protein levels of two of the candidate genes, *RORA* and *BCL-2*, were decreased in autistic brains. In this case there is a link between the methylation levels in peripheral cells and the brain. Following this study [69], whole blood DNA methylation profiles of MZ twins discordant for autism spectrum disorder (ASD) allowed the identification of DMRs associated with ASD-related traits: social autistic traits, autistic restricted repetitive behaviors and interests (RRBIs), and communication autistic traits [70]. The genome-wide study characterized 34 MZ twin pairs discordant for ASD or a related trait, 5 concordant for ASD, and 11 concordant for no autistic phenotype using the Illumina 27 K array. No global DNA methylation differences within discordant twin pairs were found, but site-specific differences were abundant. The top-ranked DMRs included genes already mentioned in the ASD literature (*GABRB3*, *AFF2*, *NLGN2*, *JMJD1C*, *SNRPN*, *SNURF*, *UBE3A*, and *KCNJ10*). Although this study explored a relatively large number of twin pairs, the findings have not yet been replicated and were based on a relatively low-coverage array (Illumina 27 K) that examines only 0.7% of variable CpG sites [88].

Pain sensitivity is a complex phenotype, and genetic effects have been unable to fully explain the variation in pain sensitivity. Bell *et al*. [79] examined DMRs associated with pain using 50 MZ twins discordant for heat pain tolerance and 50 unrelated individuals in two separate epigenome-wide association studies. Pain sensitivity was determined using the heat pain suprathreshold and DNA methylation profiles of whole blood were characterized by genome-wide MeDIP-seq. The highest association of the combined results was in the promoter of *TRPA1*, a known pain-sensitivity gene that encodes an ion channel expressed in sensory neurons, and the finding was validated using bisulfite sequencing. The observed effect at the CpG site validated by bisulfite sequencing was a 10% change in DNA methylation, which corresponded to a 2°C change in heat pain sensitivity. Pain sensitivity discordance was defined as a heat pain tolerance difference >2°C between twins. Additionally, most of the 100 top-ranked DMRs were found to be associated with genetic variants and to be stable over time. This study used a moderate sample size of 25 MZ twin pairs, combined with an unrelated sample of individuals, to identify and validate DMRs and to gain insight into underlying mechanisms of epigenetic association.

Multiple previous studies have also explored predominantly candidate genes in other complex human traits with high MZ twin discordance rates. A recent genome-wide study focused on a case of congenital renal agenesis [76]. In this study, no genetic alterations were confirmed between a pair of discordant MZ twins, but 514 DMRs were detected using reduced representation bisulfite sequencing. However, because DNA methylation differences were present in normal concordant MZ twins, further studies and larger cohorts would be needed to reach conclusive results.

The study of Down syndrome has also benefited from the use of MZ twins. Using one pair of MZ twins discordant for trisomy 21 (which causes Down syndrome) it was possible to determine that the differential expression between the twins was organized in domains along all chromosomes and that the H3K4me3 profile was altered [89]. Further study of the chromosomal arrangements caused by alterations in epigenetic states might help to provide further insights into this condition.

## Implications for disease and medicine

Twins are a unique resource for elucidating complex molecular mechanisms of disease. Epigenetic findings from disease-discordant MZ twin studies so far have identified DNA methylation changes in multiple genes across a wide range of phenotypes. In some cases, such as *DOK7* in breast cancer, the identified changes are likely biomarkers of disease but may also be involved in disease susceptibility as they pre-date the cancer diagnosis. In other examples, such as *TRPA1* in pain susceptibility, *ST6GALNAC1* in BD, *ZBTB20* in MDD, and several genes identified in ASD, the peak findings are in genes that have already been implicated in the trait. However, it remains unclear whether these changes are causal or secondary to disease, and longitudinal studies are required to address this question. However, even if the identified epigenetic changes are secondary to disease, they can still improve our understanding of disease progression, especially for relapsing disorders such as BD.

Recent findings that smoking can be reliably detected in epigenetic patterns across tissues in humans also pave the way for further use of epigenetics as an epidemiology tool - using epigenetic changes as surrogates of the environment or risk factors. The use of epigenetic markers of environmental risk would greatly improve our understanding of the molecular basis of disease, as many complex traits have an environmental risk component that is often difficult to define and assess. Therefore, using epigenetic markers of environmental disease risk would help to identify environmentally driven disease mechanisms, including gene-environment interactions.

The study of epigenetics in disease using discordant MZ twins has present and future medical implications and translational potential. Finding novel epigenetic associations with disease has implications for the identification of potential targets of drugs and biomarkers for diagnosis and prognosis. Epigenetic drugs influencing DNA methylation and chromatin modifiers are capable of reversing aberrant epigenetic states and therefore the gene regulation of abnormal cells. Examples of such drugs include the DNMT inhibitors decitabine and azacitidine for the treatment of myelodysplastic syndrome, and the combined use of a DNMT inhibitor (hydralazine) and a histone deacetylase (HDAC) inhibitor (valproate) [90]. At present, at least another 40 epigenetic drugs are reported to be in development for cancer [91].

New technologies also have the potential to allow the development of drugs for targeted epigenetic editing in the near future. Zinc-finger nucleases, transcription activator-like effector nucleases (TALENs), and the clustered regularly interspaced short palindromic repeats (CRISPRs) have allowed biological researchers to edit the genomes of various organisms [92]. The CRISPR/Cas complex, a RNA guided nuclease, is probably the most promising tool because of the relative ease with which experiments can be designed and loci targeted. The system consists of foreign DNA sequences integrated within CRISPR loci that are later transcribed. The transcript is recognized by Cas proteins and together causes the cleavage of specific sequences. The fusion of this technology with epigenetic modifiers can potentially allow targeted epigenome editing [93]. Zinc fingers coupled to the catalytic domain of Dnmt3a have been used successfully for the epigenetic reprogramming of a tumor suppressor gene (*MASPIN*) and oncogene (*SOX2*) in cancer cells [94]. Epigenetic editing targets the gene itself to silence expression, rather than targeting multiple copies of the transcript as other technologies such as RNA interference do, which makes silencing by epigenetic editing potentially more effective. Many of the findings derived from epigenetic studies could provide therapeutic targets for these types of new epigenetic drugs.

Epigenetic comparisons of MZ twins and heritability studies of DNA methylation highlight that most epigenetic variability is unique to the individual. This property makes epigenetics potentially valuable for personalized medicine. Future implications of these findings include the role of epigenetics in P4 medicine (personalized, predictive, preventive, and participative medicine), as a stable yet potentially reversible molecular mechanism.

At present, key challenges in epigenetic studies of disease-discordant MZ twins predominantly relate to sample size and power, choice and availability of appropriate tissue for the trait of interest, maximizing DNA methylation array coverage and sensitivity, and integrating epigenetic profiles with genetic, transcription, and environmental datasets (Box 1). The awareness of the value of discordant MZ studies is spreading and has led to the consolidation of international efforts, such as the EUroDiscotwin Consortium aimed at the combined collection of disease-discordant MZ twins from European and Australian Twin Registries. Furthermore, several large-scale epigenetic studies in existing twin cohorts are already under way. Whole blood is currently the sample of choice, and adjusting for cellular heterogeneity in blood samples should be a standard analytical tool in epigenetic analysis. In future, access to reference datasets of epigenetic profiles across multiple tissues will enable the establishment of tissue-specificity rates at particular epigenetic marks. As costs of sequencing technologies are constantly decreasing, reasonable genome-wide methylation coverage and sensitivity will become feasible in the near future. Lastly, methods for integrating multiple layers of functional genomic data, including epigenetic data, should be explored in the context of epigenome-wide studies of MZ discordant twins. With the use of systems biology it is increasingly possible to integrate all molecular levels of information from a patient to provide a personalized diagnosis and treatment options.

The main priorities for future research would be to explore in depth epigenetic profiles in disease-discordant MZ twins using high-resolution epigenetic assays, and replicate the findings in independent cohorts. Because twinning is a rare event, to accelerate progress large-scale efforts across international twin cohorts will be needed to reach sample sizes required to detect moderate epigenetic changes. Ultimately, longitudinal studies will be necessary to establish the timing of the epigenetic change with respect to disease onset, and to investigate its role in disease susceptibility or progression.

## Box 1. Challenges

The major challenges of epigenetics are inherent to its nature and not specific to twin studies. Epigenetic states are often tissue- and cell-specific and a sample of cells may involve a mixture of cells with different profiles. Currently, the solution to this problem is to use blood and adjust for cell sub-type counts. If blood is not available, the epigenetic states can be inferred using DNA methylation signatures from previously purified samples [95]. Another approach to overcome this difficulty is to use computational approaches, such as the FaST-LMM-EWASher method [96] or the RefFreeEWAS method [97], which adjust for cell composition without the use of reference samples. In the near future, this problem will be addressed with the use of single cell sequencing technologies based on nanopores, which can discriminate between cytosine and methylcytosine [98]. Cell composition is less of a problem for twin studies as many cell populations are genetically controlled. For example, genetic factors in twins contribute 61 to 96% of the variance in blood cells [99], and blood is the most commonly used sample for genome-wide epigenetic studies.

Related to the tissue and cell specificity of epigenetic profiles is the appropriate choice of tissue for the disease of interest. In most cases it will not be possible to study the affected tissue, such as brain in neurodegenerative diseases. Most studies use peripheral blood cells, which limits the study of epigenetic marks to those that are shared across tissues. This approach is helpful when looking for markers of the disease.

Inferring causal relationships also presents a major challenge. Associations in this field are subject to reverse causation and confounding factors. The conventional observational study at one point in time cannot determine if an epigenetic modification is a cause of the disease or if it is secondary or the result of medication use. The twin design allows us to overcome many confounding effects, but longitudinal studies are needed to determine whether an epigenetic modification precedes disease, as exemplified by Heyn *et al*. [73] in the discovery of the altered methylation status of *DOK7* before tumor diagnosis. The difficulty in these approaches is sample and phenotype collection at different time points.

The large number of different epigenetic marks is another challenge. DNA methylation is by far the most studied epigenetic mark, but histone modifications can occur in the same locus and the interaction and relative importance of different layers of epigenetic modification remain unknown. The integrative analysis of genetics, epigenetics, and transcriptomics may give a hint of the underlying regulatory mechanisms.

## Box 2. Limitations of epigenetic studies on MZ twins

Epigenetics may explain many discordances between twins, but there are limitations when dissimilarities come from other sources. Even though MZ twins were formed from the same zygote, post-zygotic mutations may occur and give rise to somatic mosaicism [51]. In some cases the mutation would be unobserved, but in others it may be the cause of a developmental disorder or may increase susceptibility to a disease in later life. Furthermore, somatic mutations continuously occur and twins may show different somatic mutation rates depending on environmental influences. The twin model assumes that MZ twins are genetically identical, but it is unknown whether these tiny differences have phenotypic consequences and to what extent.

Another potential complication is twin chorionicity, which in most cases is unknown for adult twins. Most studies treat MZ twins as a uniform group, but in fact they can be sub-classified depending on whether they shared the same placenta or not (monochorionic or dichorionic, respectively). Chorionicity is considered to be influential in epigenetic status, as reported by Kaminsky *et al*. [32], who found that DNA methylation profiles within pairs of monochorionic MZ twins are more variable than those from dichorionic MZ twins. Gordon *et al*. [45] reported similar findings and proposed that monochorionic twins are more discordant because they are more likely to compete for resources *in utero*. Both studies were small but the results are important and require further replication.

Twin chorionicity is also in part related to the potential of chimerism in MZ twins, especially when blood samples are used. *In utero*, twins can exchange fetal blood through vascular connections, and this can have implications for the detection of genetic or epigenetic events that are related to discordance originating *in utero*.

Lastly, power to detect epigenetic changes using the disease-discordant MZ twin model has not yet been fully investigated and will depend on many factors, including sample size, effect size, assay coverage and sensitivity, epigenetic heritability at the locus of interest, and longitudinal stability of the epigenetic change. Several studies have made locus-specific power estimates for the disease-discordant twin design. Kaminsky *et al.* [21] estimated reasonable power (>80% power) to detect moderate effects (1.15-fold change in methylation) using a genome-wide methylation assay targeting CpG island regulatory elements, using 21 twin pairs. However, most currently used methylation assays target methylation at single CpG sites and formal power calculations for genome-wide coverage at single CpG site resolution have not yet been reported in twins. Preliminary estimates report low (35%) to good (>80%) power to detect effects at specific CpG sites at methylation differences of 5 to 6% between affected and unaffected MZ co-twins in 20 to 22 disease-discordant twin pairs [58,71].

## Abbreviations

ASD: Autism spectrum disorder
BD: Bipolar disorder
CRISPR: Clustered regularly interspaced short palindromic repeats
DMR: Differentially methylated region
DNMT: DNA methyltransferase
DZ: Dizygotic
ICC: Intra-pair correlation coefficient
MDD: Major depressive disorder
MeDIP: Methylated DNA immunoprecipitation
MHC: Major histocompatibility complex
MZ: Monozygotic
PBMCs: Peripheral blood mononuclear cells
T1D: Type 1 diabetes
WBC: White blood cell
